# Effectiveness of Interventions and Control Measures in the Reduction of *Campylobacter* in Poultry Farms: A Comprehensive Meta-Analysis

**DOI:** 10.3390/foods15020307

**Published:** 2026-01-14

**Authors:** Odete Zefanias, Ursula Gonzales-Barron, Vasco Cadavez

**Affiliations:** 1 CIMO, La SusTEC Instituto Politécnico de Bragança, Campus de Santa Apolónia, 5300-253 Bragança, Portugal; odete.zefanias@ipb.pt (O.Z.); ubarron@ipb.pt (U.G.-B.); 2 Divisão de Ciências Animais, Instituto de Investigação Agrária de Moçambique (IIAM), Av. de Moçambique, Km 1.5, Maputo 1922, Mozambique

**Keywords:** campylobacteriosis, primary production, chicken farms, feed additives, probiotics, bacteriophages, plant extracts, litter management, environmental reservoirs, biosecurity, multilevel modelling, meta-regression

## Abstract

*Campylobacter* is a leading foodborne bacterial pathogen, and poultry production is a major reservoir contributing to human exposure. Reducing *Campylobacter* at farm level is therefore critical to limit downstream contamination. This systematic review and meta-analysis aimed to identify and quantitively summarise the current interventions and control measures applied in poultry farms to control the contamination and bird colonisation by *Campylobacter*. The Scopus electronic database was accessed to collect primary research articles that focused on observational studies and in vivo experiments, reporting results on *Campylobacter* concentrations or prevalence in both non-intervened and intervened groups. A total of 4080 studies were reviewed, from which 112 were selected and included in the meta-analysis according to predefined criteria, yielding 1467 observations. Meta-regression models were adjusted to the full data set and by intervention strategy based on the type of outcome measure (i.e., concentration and prevalence). In general terms, the results reveal that the effectiveness to reduce *Campylobacter* colonisation vary among interventions. A highly significant effect (*p* < 0.001) was observed in interventions such as organic acids, bacteriophages, plant extracts, probiotics, and organic iron complexes added to feed or drinking water; although drinking water was proven to be a more effective means of administration than feed for extracts and organic acids. In contrast, interventions such as chemical treatments, routine cleaning and disinfection, and vaccination showed both lower and more heterogeneous effects on *Campylobacter* loads. Vaccination effects were demonstrated to be driven by route and schedule, with intramuscular administration, longer vaccination periods and sufficient time before slaughter linked to greater reduction in *Campylobacter* colonisation. Probiotics, plant extracts and routine cleaning and disinfection were associated with lower *Campylobacter* prevalence in flocks. Meta-regression models consistently showed that the interventions were proven more effective when the sample analysed was caecal contents in comparison to faeces (*p* < 0.001). Overall, the findings of this meta-analysis study emphasise the application of a multi-barrier approach that combines targeted interventions with robust biosecurity and hygiene measures in order to reduce *Campylobacter* levels in poultry farms.

## 1. Introduction

*Campylobacter* infection in poultry production is a global concern, with prevalence and incidence varying between countries [1]. Owing to its high prevalence in broiler flocks and poultry meat, the European Food Safety Authority (EFSA) has classified *Campylobacter* as a public health priority hazard in poultry meat inspection [2]. In the European Union, campylobacteriosis remains the most frequently reported zoonosis; in 2023, 148,181 human cases were reported (45.7 cases per 100,000 population) [3]. Beyond morbidity, the economic burden is substantial, with the overall annual cost to public health systems and lost productivity in the EU estimated at around EUR 2.4 billion [4]. Although *Campylobacter* is frequently detected in poultry, the relative contribution of different sources and transmission routes in primary production is not yet fully understood [5]. Recent One Health evidence has reported *Campylobacter jejuni* in broilers and laying hens as well as in poultry farmers, underscoring the relevance of occupational exposure and the need to better characterise transmission pathways in primary production [6]. Horizontal transmission, in particular, has been identified as a major pathway for introducing *Campylobacter* into poultry flocks [1].

The presence of *Campylobacter* in the farm environment poses significant risks for its introduction into poultry houses [1,7]. Key risk factors that can facilitate transmission in chicken flocks include untreated drinking water, reused or insufficiently disinfected litters, vectors such as insects and rodents, farm workers, contaminated equipment and utensils, and other nearby animal species such as pigs, cattle and pets [8]. *Campylobacter* occurrence is also influenced by flock and management characteristics, including breed, production system (conventional, free-range, organic) and flock size [9,10]. Moreover, infection dynamics vary with bird age [11]; several studies have reported that chicks are usually free of *Campylobacter* up to approximately 14 days of age [5,12], a pattern often attributed to maternal immunity and the immature gut environment in early life [13,14].

Risk assessments indicate that reducing *Campylobacter* loads in the poultry meat production chain by about 3 log CFU/g could decrease the risk to public health by roughly 90% [15]. To achieve such reductions, it is necessary to understand *Campylobacter* prevalence and bacterial loads at different stages of production and processing, and to identify critical control points along the farm-to-slaughter continuum [8]. Historically, antibiotics have been used in livestock production not only to treat and prevent disease but also as growth promoters [16]. However, the rise in antimicrobial resistance among foodborne bacteria has led to regulatory restrictions and a strong push towards non-antibiotic interventions [17,18]. Similar relationships between farm practices, antimicrobial usage, and antimicrobial resistance have also been documented in other livestock systems, including pig farms [19]. As a result, a range of on-farm measures has been investigated, including routine cleaning and disinfection, feed and drinking-water additives such as organic acids, and other alternatives such as plant extracts, probiotics, prebiotics, bacteriophages and vaccines [20,21,22]. Nevertheless, no single intervention has proven fully effective in consistently reducing *Campylobacter* contamination in poultry, and their effects appear to depend on factors such as dose, timing of application, production system and local conditions [20].

Although the literature presents numerous reviews focused on sources or intervention strategies to control *Campylobacter* on poultry farms, these studies lack a quantitative synthesis that relates the interventions and/or moderators.

Therefore, this study aimed to conduct a systematic review and meta-analysis to (i) identify the on-farm interventions and control measures that can be implemented in poultry production to control *Campylobacter*; (ii) quantify their impact on *Campylobacter* concentration and prevalence in poultry flocks; and (iii) determine the main environmental and management-related risk factors that contribute to the spread of *Campylobacter* in poultry farms.

## 2. Materials and Methods

This work is a systematic review and meta-analysis, that is, a structured synthesis of primary studies that applies explicit methods to minimise bias and, when possible, statistically combines effect sizes from independent studies into pooled estimates [23,24,25].

### 2.1. Study Design and Research Question

The systematic review was conducted in accordance with the Preferred Reporting Items for Systematic Reviews and Meta-Analyses (PRISMA) guidelines [26]. The research question was formulated using the Population, Intervention, Comparator, and Outcome (PICO) framework [27].

The **population** comprised poultry (chickens, turkeys and ducks) raised on any type of farm and in any country. **Interventions** included any on-farm intervention, treatment or control measure implemented in the primary production with the aim (explicitly or implicitly) of influencing *Campylobacter* colonisation, without restrictions on the type of intervention. The **comparator** was a concurrent non-intervened or non-treated group (i.e., control) within the same study. The **outcomes** considered were *Campylobacter* concentration and prevalence in samples collected from the control and treated groups, as well as environmental samples when reported.

### 2.2. Literature Search Strategy

A targeted literature search was performed in July 2024 using the Scopus electronic database to identify original articles published from 1995 onwards. Searches were carried out in the title, abstract and keywords fields. Bibliographic searches combined terms related to *Campylobacter* infection or colonisation in poultry, on-farm interventions, control measures, treatments or risk factors, connected by the logical operators “AND” and “OR”.

The following search string was employed:

((prevalence) OR (“risk factor”) OR (“control measures”) OR (“control measure”) OR (risk) OR (intervention*) OR (strateg*) OR (“observational study”) OR (“cohort”) OR (“longitudinal study”) OR (cross-sectional) OR (“cross sectional”) OR (farm*) OR (source*) OR (colonisation) OR (colonization) OR (“control measure”) OR (“control measures”) OR (husbandry) OR (management) OR (“free range”) OR (free-range) OR (slaughter*)) AND ((“avian campylobacteriosis”) OR (“campylobacter infection”) OR (campylobacter) OR (campylobacteriosis)) AND ((poultry) OR (broiler) OR (hen*) OR (chicken*) OR (chick*) OR (duck) OR (turkey) OR (bird*) OR (flock*)).

### 2.3. Eligibility and Exclusion Criteria

All records retrieved from Scopus were imported into Rayyan software 1.4 [28] for screening. Since only one database was used, no duplicate records were identified. Titles and abstracts were independently screened by two reviewers according to predefined eligibility criteria. Studies were included if they met all of the following conditions: (i) written in English, Spanish or Portuguese; (ii) primary, original research; (iii) conducted in poultry (chickens, turkeys or ducks) under farm conditions (including experimental farms); (iv) reported on farm-level interventions, control measures or risk factors in the poultry environment and/or in vivo interventions or treatments in poultry aimed at controlling *Campylobacter*; and (v) provided data on *Campylobacter* concentration and/or prevalence in both control and treated groups (or in defined environmental sources), allowing subsequent calculation of effect sizes for prevalence (risk ratio) and counts (log reduction) associated with the intervention or risk factor.

No restrictions were applied regarding country or type of intervention. Studies were excluded if: (i) no intervention to control *Campylobacter* or no explicit environmental risk factor was evaluated; (ii) no sufficient data were provided to calculate the effect size for *Campylobacter* concentration or prevalence; (iii) the primary focus was antimicrobial resistance in *Campylobacter*; or (iv) the article was a review, systematic review or meta-analysis.

To minimise the influence of clearly biased evidence, we further excluded studies with obvious problems in selection, methodology, detection or reporting quality. Examples included: non-random or diseased flocks without appropriate controls (selection bias), insufficient description or referencing of laboratory methods (methodological bias), missing key analytical details such as sample weight (detection bias), and inconsistencies between results reported in the text and in tables or figures (reporting bias). Only studies that were not assigned any bias were retained for quantitative synthesis. We did not apply a formal risk-of-bias tool (e.g., ROBINS-I), which should be considered a limitation of this review.

### 2.4. Data Extraction

After the preliminary screening, full texts of eligible articles were retrieved and relevant data were extracted manually into a structured Microsoft Excel spreadsheet. Data extraction was performed by one reviewer and independently checked by two reviewers. Any discrepancies were resolved through discussion and consensus; when required, a third reviewer was adjudicated. The following information was recorded for each study:

(a) **Study identification**: Reference code and full citation.
(b) **Country**: Country in which the study was conducted.
(c) **Bacterial species**: *Campylobacter* species investigated.
(d) **Bird species**: Chickens, turkeys or ducks.
(e) **Bird age at baseline**: Age of birds at the beginning of the experiment.
(f) **Intervention type**: Tested interventions such as bacteriophages, probiotics, prebiotics, organic acids, plant extracts, or chemical products. This field also encompassed control measures and risk factors such as routine cleaning and disinfection practices (before and after), production system (conventional versus free-range), season, and transport of birds to slaughterhouses (before and after).
(g) **Application matrix**: Route of administration, including incorporation into drinking water, feed or litter, or direct delivery to birds via oral gavage, intramuscular or subcutaneous injection, or spraying.
(h) **Timing and duration of treatment**: Age at the start of application and duration of treatment (days).
(i) **Applied dose**: Concentration or dose of the intervention as reported by the authors.
(j) **Slaughter age**: Age of birds at slaughter.
(k) **Sample type**: Faeces, caeca, carcasses, water, soil, environmental swabs, litter, floor and other matrices, as reported.
(l) **Sample weight or volume**: Employed for *Campylobacter* analysis either detection or enumeration.
(m) **Counts results**: Sample size (total number of samples), measures of central tendency (mean and/or median concentration in log CFU/g or log CFU/mL) and standard deviations for both control and treatment groups. When standard error of the mean was reported, the standard deviation was derived accordingly.
(n) **Prevalence results**: Sample size (total number of samples) and number of positive samples for both control and treatment groups.
  For studies in which birds were experimentally infected with *Campylobacter*, we additionally extracted:
(o) **Inoculation details**: Inoculum size (log CFU/g or log CFU/mL), volume administered (mL) and age at infection.
  For the analysis of *Campylobacter* in environmental sources, we additionally extracted:
(p) **Sample source**: Air, boot socks, live animals, deep litter, drinkers, feeders, farm operators, floor faeces, insects or other environmental sources.

When results were only presented graphically, numerical data were extracted using Plot Digitizer software version 5.2 [29].

### 2.5. Meta-Regression Models

The meta-analysis study considered three main effect sizes depending on the type of outcome variable: (i) an effect size measured as the log reduction attained by on-farm interventions using *Campylobacter* counts data; (ii) an effect size measured as the log risk ratio of on-farm interventions using *Campylobacter* prevalence data; and (iii) an effect size measured as the logit proportion of the presence of *Campylobacter* in live birds and environmental samples from poultry farms. For clarity, the logit proportion is the log-odds transformation of a proportion *p*, defined as ln(*p*/(1 − *p*)) [30].

For concentration outcomes, each effect size was calculated as log reduction, that is, the difference between the mean log_10_ concentration in the control and treated groups:

$$y_{i}=C_{control\;i}-C_{treated\;i}$$

where $C_{control\;i}$ and $C_{treated\;i}$ are the mean *Campylobacter* concentrations (log_10_ CFU/g or log_10_ CFU/mL) in the study i for the control and treated groups, respectively. Positive values of $y_{i}$ indicate a reduction in concentration in the treated group, whereas negative values indicate higher concentrations in the treated group.

Three types of random-effects meta-regression models were fitted to the log reduction effect size data: (i) a meta-regression model utilising the complete data set, placing intervention as moderator, in order to summarise the pooled mean log reduction in *Campylobacter* load attained by the different interventions; (ii) univariate meta-regressions separately fitted by intervention, in which quantitative moderators were entered to the model one at a time to explore their association with the log reduction. The moderators included slaughter age, duration of treatment, age at application, applied dose, inoculum level at experimental challenge and age at challenge; and (iii) multivariable meta-regressions separately fitted by intervention, in which several moderators were now included simultaneously to determine the most important ones driving the reductions in *Campylobacter* load.

For prevalence outcomes, the effect size $y_{i}$ of interventions comparing treated and control groups was expressed as the natural logarithm of the risk ratio (RR), defined as

$$y_{i}={RR}_{i}=ln\frac{p_{control\;i}}{p_{treated\;i}}$$

where $p_{control\;i}$ and $p_{treated\;i}$ are the proportions of *Campylobacter*-positive samples in the control and treated groups, respectively, for study *i*. Values of $y_{i}$ greater than zero indicate a reduction in prevalence in the treated group relative to the control.

Two types of random-effects meta-regression models were fitted to the log risk ratio effect size data: (i) an overall meta-regression model utilising the complete data set, placing intervention as moderator, in order to summarise the pooled log reduction in *Campylobacter* prevalence attained by the different interventions; and (ii) multivariable meta-regressions separately fitted by intervention, in which several moderators were included simultaneously to determine the most important one driving the reductions in *Campylobacter* prevalence.

For single-group prevalence outcomes relative to live birds and on-farm environmental sources (i.e., litter, floor faeces, air, insects), the effect size $y_{i}$ was defined as the logit transformation of the proportion of positive samples ($p_{i}$) for each source *i*,

$$y_{i}=\ln\frac{p_{i}}{\left(1-p_{i}\right)}$$

A meta-regression model was fitted to the logit proportion data set, placing source as moderator, in order to summarise the overall *Campylobacter* prevalence in live birds and environmental sources. In all meta-regressions, effect sizes were weighted by the inverse of their sampling variance [31,32].

For the three type of effect sizes described above, random-effects meta-regression models had the following general form:

$$y_{ij}=\beta_{0}+\sum_{k}\left(\beta_{k}\times x_{k\;ij}\right)+\;u_{i}+\;\varepsilon_{ij}$$

where $y_{ij}$ is the effect size for observation *j* belonging to study *i*, $x_{k\;ij}$ are qualitative or quantitative moderator variables, $\beta_{k}$ are fixed-effect coefficients, $u_{i}$ is the study-level random effect with variance τ^2^, and $\varepsilon_{ij}$ is the within-study sampling error. All statistical analyses were performed using R software version 2025. 5.0 [33], using the metafor package for all meta-regressions and graphs [34], and sqldf for data handling, subsetting and querying [35].

### 2.6. Assessment of Heterogeneity, Accuracy and Consistency, and Publication Bias

Heterogeneity and publication bias were assessed for the overall and the multivariate meta-regression models. Between-study heterogeneity refers to the variability in the true intervention effects across studies, beyond what would be expected by sampling error alone [36]. To quantify the impact of this between-study variability, we used the I^2^ statistic, which expresses the percentage of total variability in observed effect sizes that is attributable to heterogeneity rather than chance [32,37].

From a null random-effects model including only an intercept, we obtained the within-study variance (s^2^) and the between-study variance (τ^2^). The I^2^ statistic was then calculated as

$$I^{2}=\frac{\tau^{2}}{\left(\tau^{2}+s^{2}\right)}\times 100\%$$

A value of 0% can be interpreted as no observed heterogeneity, whereas values of ~25%, 50% and 75% are interpreted as low, moderate and high heterogeneity, respectively [32,38]. After fitting meta-regression models with moderators, we obtained the residual between-study variance ($\tau_{res}^{2}$). The proportion of between-study variance explained by the set of moderators was summarised using an R^2^ measure:

$$R^{2}=\frac{\left(\tau^{2}-\tau_{res}^{2}\right)}{\tau^{2}}$$

where higher values indicate that a larger fraction of the heterogeneity is accounted for by the moderator(s) included in the model.

The accuracy and consistency of the study results were explored using Galbraith (radial) plots [39,40]. In these plots, the standardised effect size $z_{i}$ for each observation *i* ($z_{i}=y_{i}/{SE}_{i}$) is plotted on the y-axis against precision ($1/{SE}_{i}$) on the *x*-axis (SE: standard error). Under a homogeneous random-effects model, points are expected to fall within approximate 95% reference bands around the regression line. Deviations from this pattern and points lying far outside the reference bands are used to visually assess heterogeneity; and to identify potential outliers.

Uncertainty around pooled effects and moderator coefficients was summarised through the standard errors reported in the tables, associated *p*-values, and the confidence intervals linked to these statistics. Galbraith plots provided a complementary graphical assessment of consistency and possible influential observations.

Publication bias arises when studies reporting larger or more “positive” effects are more likely to be published than studies with smaller or null effects, which can distort pooled estimates [36]. In every meta-regression model, potential publication bias and small-study effects were evaluated using two approaches. First, funnel plots were inspected, in which effect sizes are plotted against their standard errors: in the absence of bias, the points are expected to form an approximately symmetrical, inverted funnel, whereas marked asymmetry may indicate publication bias or other small-study effects [25,36]. Secondly, we explored the relationship between effect size and study size by including the total sample size as a moderator in meta-regression models [41]. A statistically significant association between sample size and the effect size (*p* < 0.05) was interpreted as suggestive evidence of small-study effects that could be compatible with publication bias, and was considered alongside the funnel plot patterns.

## 3. Results and Discussion

### 3.1. Description of the Meta-Analytical Data

**Figure 1:**
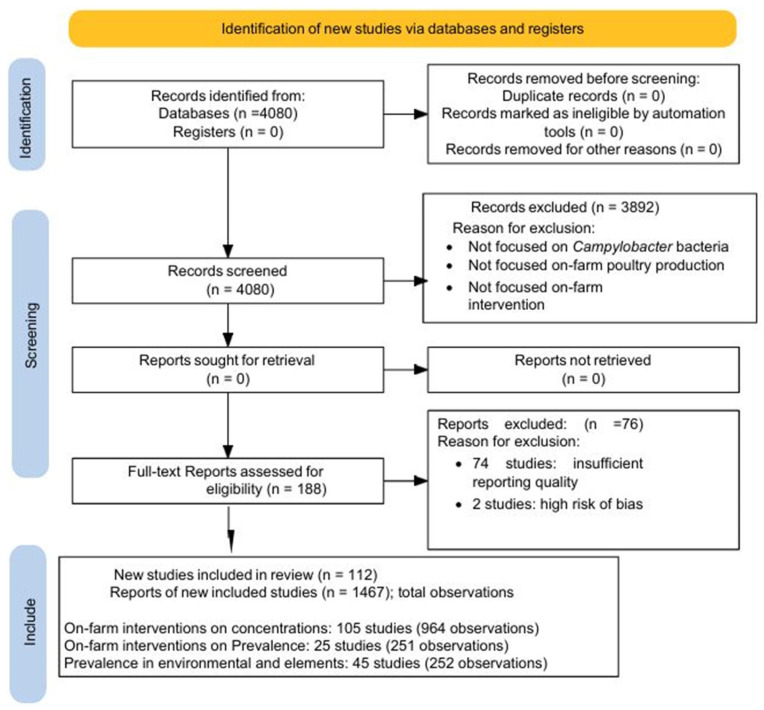
Flowchart illustrates the article selection process for the systematic review and meta-analysis of interventions, control measures and treatments, aimed at controlling *Campylobacter* in poultry farms.

The steps of the study selection process for this meta-analysis are summarised in the PRISMA flow diagram (Figure 1). A total of 4080 records were retrieved from the Scopus database. After screening titles, abstracts and keywords according to the predefined inclusion and exclusion criteria, 188 articles were retained for full-text assessment, and 3892 records were excluded. Data were extracted from all 188 articles following complete reading. Of these, 112 articles met the criteria for inclusion in the quantitative synthesis (meta-analysis), whereas the other 76 studies were excluded because effect sizes could not be reliably derived or because of insufficient statistical reporting and other methodological concerns, including suspected bias. In total, 1467 effect-size observations were available for analysis. Although the review covered chickens, turkeys and ducks, most intervention studies were conducted in chickens. Effect size observations in chickens accounted for 97% of the data.

The meta-analysis of on-farm interventions in relation to *Campylobacter* concentration in poultry was based on 105 studies contributing 964 observations [42,43,44,45,46,47,48,49,50,51,52,53,54,55,56,57,58,59,60,61,62,63,64,65,66,67,68,69,70,71,72,73,74,75,76,77,78,79,80,81,82,83,84,85,86,87,88,89,90,91,92,93,94,95,96,97,98,99,100,101,102,103,104,105,106,107,108,109,110,111,112,113,114,115,116,117,118,119,120,121,122,123,124,125,126,127,128,129,130,131,132]. On-farm interventions in relation to *Campylobacter* prevalence in poultry were analysed using 25 studies with 251 observations [52,62,73,76,78,81,94,97,106,107,121,123,130,131,133,134,135,136,137,138,139,140,141,142]. Finally, the analysis of on-farm prevalence of *Campylobacter* in live animals and environmental elements was based on 45 studies with 252 observations [141,143,144,145,146,147,148,149,150,151,152,153,154]. Some studies contributed data to more than one of these outcome categories.

### 3.2. Summarisation of the Effects of On-Farm Interventions and Treatments on the Concentration of Campylobacter in Poultry

To assess the overall effects of on-farm interventions and treatments on *Campylobacter* load in poultry farms, random-effects meta-regression models were fitted to the pooled concentration data. Pooled effect sizes of log reduction, defined as the difference between the mean log concentration in the control and treated groups, are compiled in Table 1, with positive values indicating lower *Campylobacter* concentrations in treated birds.

For each intervention type, the meta-regression model also estimated the standard error and *p*-value, which is useful to compare the degree of effectiveness of the interventions. Nonetheless, non-significance can also arise from lack of agreement (i.e., high heterogeneity) between studies.

Overall, the overall meta-regression model showed a statistically significant reduction in *Campylobacter* concentration for organic acids in feed/water, bacteriophages in feed/water, plant extracts in feed/water, probiotics and prebiotics in feed/water (*p* < 0.001; Table 1). Among these, organic acids, bacteriophages and plant extracts produced reductions in caecal/faecal contents of approximately 0.7–0.9 log units, while organic iron complexes showed the largest estimated reduction (1.599 ± 0.886 log), although this effect did not reach conventional statistical significance (*p* = 0.071), arisen from the small number of studies. In contrast, no significant effects on *Campylobacter* concentration were observed for chemical treatments in feed/water/litter (*p* = 0.112), routine cleaning and disinfection (*p* = 0.236), vaccination (*p* = 0.674) or for the comparison between conventional and alternative production systems (*p* = 0.895). Although not statistically significant (*p* = 0.573), transport of birds to slaughterhouses was associated with a slight increase in *Campylobacter* concentration.

**Figure 2:**
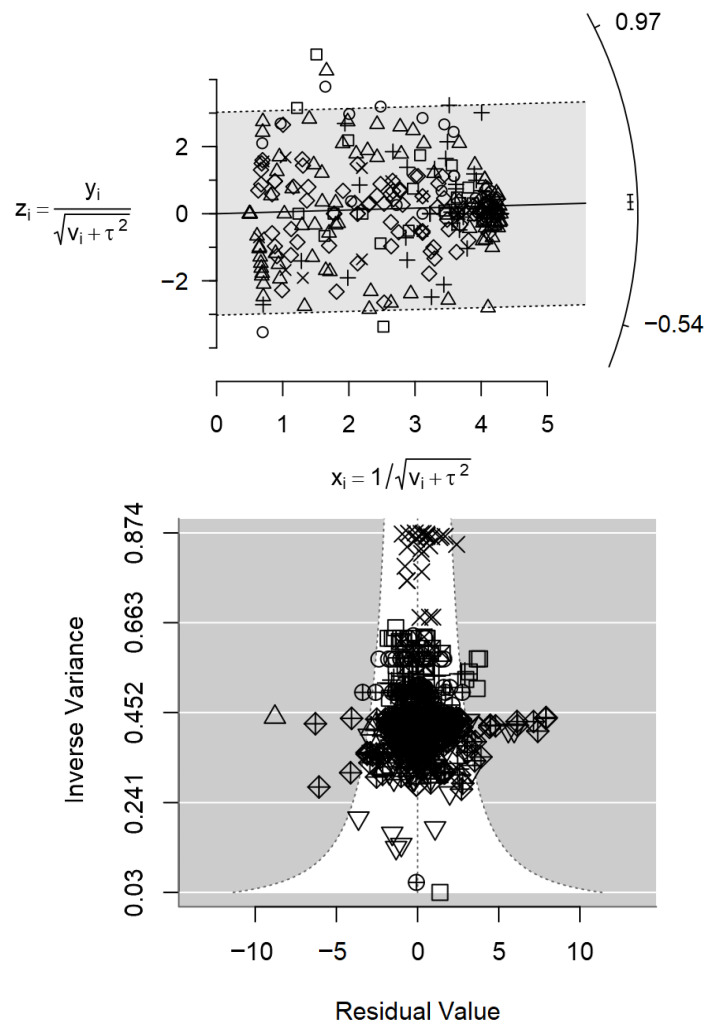
Galbraith (radial) plot (**top**) and funnel plot (**bottom**) for the overall random-effects meta-analysis of the effects of on-farm interventions on *Campylobacter* concentration in poultry. Symbols represent different intervention categories, including □ = *Cleaning and disinfection*, ○ = *Transport*, ∆ = *Bacteriophages*, + = *Chemical treatment*, × = *Non-conventional production systems*, ◊ = *Extracts*, 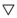 = *Organic acids*, 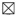 = *Organic iron complex*, * = *Prebiotic*, 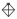 = *Probiotic*, 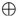 = *Vaccination*.

The Galbraith plot in Figure 2 (top) shows that most standardised effect sizes lie within the 95% confidence interval bands, with only a few outlying observations, suggesting generally consistent results across the range of values. Only a small number of observations lay outside the outer reference lines, which is consistent with the established condition of excluding studies with obvious methodological or reporting problems. Outliers were not removed from the analysis.

However, the regression of effect size on study size indicated evidence of small-study effects compatible with publication bias (*p* < 0.001; Table 1), which is consistent with the slight asymmetry observed in the funnel plot (Figure 2, bottom). Despite the high number of primary studies synthesised, the overall meta-regression analysis revealed only moderate between-study heterogeneity (I^2^ = 54.3%). The inclusion of all interventions in the multivariable model explained about one-third of this heterogeneity (R^2^ = 32%), although some residual heterogeneity remained (Q-test for residual heterogeneity, *p* < 0.001).

### 3.3. Univariate Analysis of On-Farm Interventions and Treatments on the Concentration of Campylobacter in Poultry

To estimate the influence of each quantitative moderator on *Campylobacter* concentration, a series of univariable random-effects meta-regression models was fitted (Table 2). Each model included a single quantitative variable at a time to evaluate how slaughter age, duration of treatment, age at application, applied dose, inoculum level at experimental challenge and age at challenge were associated with the log reduction in *Campylobacter* concentration.

Through this analysis, many potential relationships between quantitative moderators and intervention effects could be elucidated. For the intervention of bacteriophages-added feed, the estimated log reduction tended to decrease with increasing bird age, suggesting that bacteriophages could be less effective in reducing *Campylobacter* concentrations in older birds.

**Figure 3:**
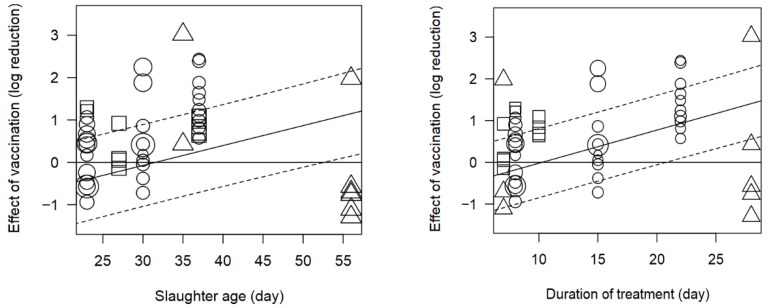
Meta-analytical bubble plots depicting the impact of (*p* < 0.10) slaughter age (**left**) and duration of treatment (**right**) on the log reduction in *Campylobacter* in poultry due to vaccination. Marker size represents study’s weight, which is the reciprocal of the study’s variance. Left and right: □ = *Intramuscular*, ○ = *Oral gavage*, ∆ = *Subcutaneous*.

The age at slaughter had a significant (α < 0.10) and positive influence on the reduction in *Campylobacter* concentration when organic acids, prebiotics, chemical treatments and vaccines were used (Table 2), indicating that these interventions tended to achieve larger log reductions when used in birds closer to slaughter. In contrast, for bacteriophages and probiotics, the significant slopes were negative, meaning that the estimated effect weakened with increasing slaughter age and, in some cases, moved towards higher *Campylobacter* concentrations in older birds. These age-related differences in treatment efficacy are likely related to factors such as microbial adaptation, changes in colonisation sites along the gastrointestinal tract, the maturation of the gut microbiota and immune system [155], and differences in the persistence of each intervention [156,157]. In line with this, vaccines appeared to be most effective in birds slaughtered between 30 and 40 days of age, whereas the effect was less noticeable between 20 and 30 days, as illustrated by the bubble plot in Figure 3 (left).

**Figure 4:**
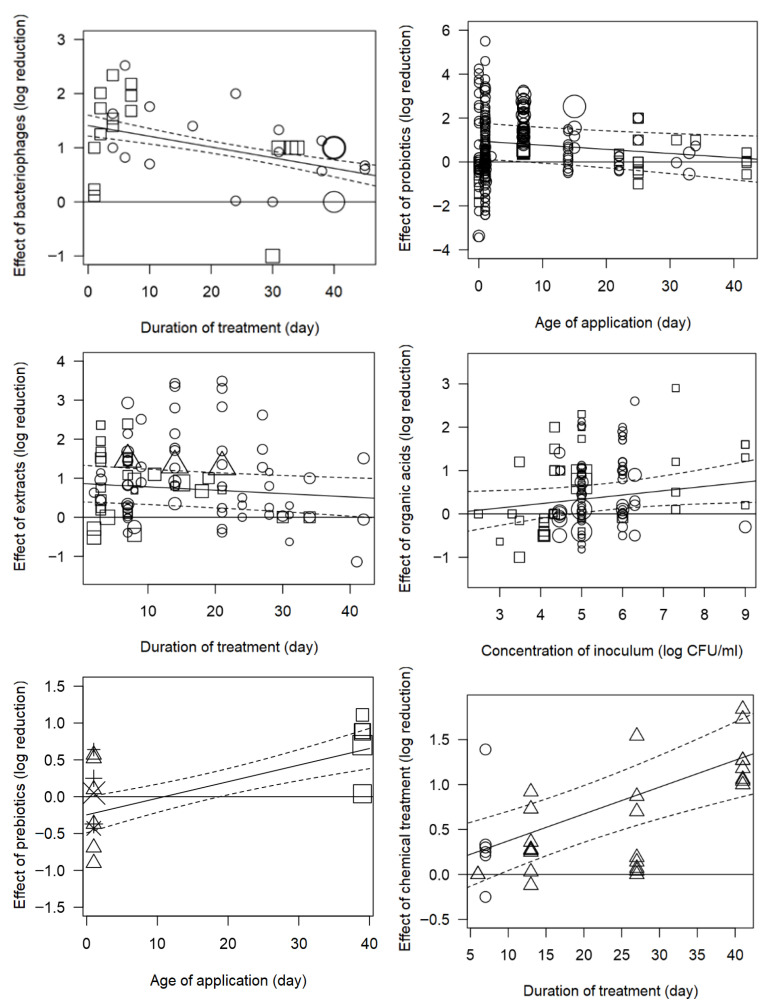
Meta-analytic bubble plots showing the significant (*p* < 0.10) relationships between quantitative moderators and the effects of on-farm interventions on *Campylobacter* concentration in poultry. Bubble size is proportional to the weight of each observation in the meta-regression models. Marker size represents study’s weight, which is the reciprocal of the study’s variance. Top left and right: □ = *Faeces*, ○ = *Caecal*; Middle left: □ = *Faeces*, ○ = *Caecal*, ∆ = *Water*; Middle right: □ = *Drinking water*, ○ = *Feed*; Bottom left: □ = *Fructooligosaccharides*, ∆ = *Galactooligosaccharides*, + = *Mannanoligosaccharides*, × = *Bacitracin methylene disalicylate*; Bottom right: ○ = *Feed*, ∆ = *Litter treatment*.

With respect to the duration of treatment, univariate models indicated that increasing the number of days of bacteriophage application was associated with smaller reductions in *Campylobacter* concentration (Table 2). The bubble plots in Figure 4 (top left) show that bacteriophages tended to produce marked short-term reductions in younger birds, but these effects diminished in older birds and as time since application increased. This pattern is consistent with previous work in which bacteriophages administered at around 25 days of age produced substantial reductions within the first 24 h, but their efficacy declined after 3 days, with considerable variability between birds [158]. These findings suggest that bacteriophages may be most effective when applied relatively close to slaughter and in younger birds, and that their effects may be transient, although the number of available studies remains limited.

The effect of plant extracts in feed also depended on the treatment duration (Table 2). Reductions in *Campylobacter* load were most pronounced for treatment periods of approximately 5–21 days and tended to decrease with longer durations, as shown by the bubble plots in Figure 4 (middle left). In contrast, treatment duration was positively associated with the effects of probiotics, chemical treatments and vaccines, suggesting that longer application periods were linked to greater reductions in *Campylobacter* load. This pattern is particularly evident for vaccines in Figure 3 (right).

Regarding age at application, the univariate analysis (Table 2) showed a negative association between bird’s age at application and the effect of probiotics, meaning that their ability to reduce *Campylobacter* colonisation decreased when they were introduced later in life. The bubble plots in Figure 4 (top right) indicate that probiotics were more effective when administered to younger birds, with diminishing effects as age increased. Conversely, age at application was positively associated with the effect of prebiotics, which showed greater reductions in older birds, as illustrated in Figure 4 (bottom left).

The dose applied in feed/water also influenced treatment efficacy. Higher doses of probiotics, plant extracts, organic acids and organic iron complexes were generally associated with larger reductions in *Campylobacter* concentration (Table 2). For bacteriophages, however, the relationship with dose was inverse, suggesting that higher phage doses were not necessarily accompanied by greater reductions and may even have been used in contexts (e.g., older birds or heavily colonised flocks) where apparent efficacy was lower. Given the small number of studies for some interventions, these dose–response patterns should be interpreted with caution.

The inoculum level used in experimental challenge studies (log CFU/mL) also moderated intervention effects. Univariate analyses indicated that higher *Campylobacter* challenge doses inoculated to chickens were associated with a reduced efficacy of probiotics, whereas the performance of organic acids was less affected (Table 2). This pattern suggests that very high challenge doses may mask or overwhelm the protective effect of probiotics, whereas organic acids remained effective over a wider range of inoculum levels. For example, Figure 4 (middle right) shows that organic acids achieved substantial reductions at inoculum concentrations between approximately 4.5 and 7 log CFU/mL.

Finally, the age at which birds were experimentally infected with the *Campylobacter* strain influenced the measured impact of plant extracts and vaccines on *Campylobacter* colonisation. Vaccines tended to be less effective than plant extracts under late challenge conditions (Table 2). This can be explained by the fact that most *Campylobacter* vaccines for chickens are designed to target the early colonisation window, around 14 days of age, when maternal antibodies wane and colonisation are just beginning [159]. Vaccination at later ages, when *Campylobacter* is already well established in the gut microbiota, is less likely to prevent or clear colonisation, and protective immunity may not fully develop if immunisation occurs after colonisation [159,160]. Thus, the available data suggest that late vaccination of already colonised older birds is unlikely to result in marked reductions in *Campylobacter* loads, whereas early interventions and plant extracts may retain some efficacy.

### 3.4. Multilevel Meta-Analysis on the Effect On-Farm Interventions on the Concentration of Campylobacter in Poultry

Given the substantial between-study heterogeneity and indications of small-study effects in the overall meta-regression model, multilevel meta-regression analyses were fitted separately for each intervention category to better understand the influence of study-level moderators on *Campylobacter* loads.

The best-fitting multilevel models for bacteriophages, probiotics, extract plants, organic acids and prebiotics interventions, including the significant moderators retained, the heterogeneity analysis and the publication bias assessment, are summarised in Table 3.

#### 3.4.1. Effectiveness of Bacteriophages Against *Campylobacter* Colonisation Levels

For bacteriophage interventions, the multilevel meta-regression identified treatment duration, applied dose, age at application and sample type (caeca versus faeces) as significant moderators (*p* < 0.05; Table 3). As in the univariate analyses, the multilevel meta-analysis reinforced that treatment duration has a negative association with the log reduction in *Campylobacter* colonisation levels (−0.021 ± 0.005; *p* = 0.001), indicating that longer bacteriophage application periods were associated with smaller reductions. This suggests that the effect of bacteriophages is largely short-lived and may decline over time, which is consistent with concerns about the potential emergence of bacteriophage-resistant bacterial mutants [158]. In line with previous work, the literature recommends applying bacteriophages shortly before slaughter, for example, two days pre-slaughter to maximise their effect [158]. The applied dose also showed an inverse association with the estimated effect (−0.041 ± 0.010; *p* < 0.001), implying that higher reported bacteriophage doses were not consistently associated with larger reductions in *Campylobacter* concentration. This pattern should be interpreted with caution, as higher doses were often used in more heavily colonised or older flocks, where responses may have been weaker.

In contrast, age at application of treatment had a small but positive significant coefficient (0.015 ± 0.005; *p* = 0.017), suggesting that, within the range of ages studied and after accounting for other moderators, applications at slightly older ages were associated with greater log reductions. This is broadly compatible with other meta-analyses indicating that bacteriophage treatments can be effective when administered close to the slaughter age [161]. Sample type also influenced the estimated effect: the coefficient for caecal samples (0.386 ± 0.161; *p* = 0.017) indicates that effect sizes measured across the primary studies were impacted by how colonisation levels were quantified, either in caecal content or in faeces. Differences between caecal contents and faecal samples are biologically plausible and methodologically expected. The caeca are the primary intestinal niche for *Campylobacter* colonisation in poultry, where bacterial loads are typically highest and more directly reflect true intestinal colonisation. In contrast, faecal measurements reflect shedding and are influenced by gut transit, dilution and mixing across intestinal compartments, as well as environmental exposure after excretion (oxygen, moisture and temperature) and heterogeneous sampling (e.g., droppings collected from litter/floor). These factors can increase variability and measurement error in faecal counts and prevalence; potentially attenuating or obscuring intervention effects compared with caecal sampling. Additionally, matrix effects and limits of detection/quantification may differ between caecal and faecal material, further contributing to systematic differences in estimated effects across studies. [162,163]. More broadly, bacteriophages have been highlighted as a promising non-antibiotic approach to combat foodborne pathogens, including biofilm-associated contamination, supporting continued development of phage-based interventions [164].

For bacteriophage interventions, between-study heterogeneity was low (I^2^ = 14%), and the moderators retained in the model explained nearly half the between-study variance (R^2^ = 43%). Some residual heterogeneity remained (Q_E test, *p* < 0.001), which may reflect unmeasured study-level differences such as flock management, background microbiota or phage cocktail composition. The test for small-study effects did not provide evidence of publication bias (*p* = 0.407), suggesting a relatively consistent pattern of results across studies [38].

#### 3.4.2. Effectiveness of Probiotics Against *Campylobacter* Colonisation Levels

For the intervention of probiotics in feed, the multilevel meta-regression model retained treatment duration, age at application, inoculum concentration and sample type (caeca versus faeces) as quantitative and categorical moderators. All these variables showed statistically significant or borderline associations with the estimated log reduction in *Campylobacter* colonisation levels (*p* ≤ 0.057; Table 3).

Treatment duration was positively associated with the effect of probiotics (0.012 ± 0.002; *p* < 0.001), indicating that longer probiotic administration was linked to greater reductions in *Campylobacter* concentration. This pattern is consistent with the notion that sustained administration allows beneficial microbiota to establish and persist, thereby enhancing competitive exclusion of *Campylobacter* through mechanisms such as nutrient competition, production of antimicrobial compounds and modulation of gut immunity [165].

Age at application showed a negative association (−0.025 ± 0.009; *p* = 0.007), meaning that the later the probiotics are introduced, the smaller the observed reduction in *Campylobacter* concentration. In practical terms, this suggests that probiotics are more effective when administered early in life, ideally from hatching, and that their efficacy decreases when administration is delayed, likely because *Campylobacter* is already established in the gut and the intestinal ecosystem is less amenable to modulation [155,157,165].

Inoculum concentration also showed a negative association with the probiotic effect (−0.319 ± 0.168; *p* = 0.057), indicating that higher *Campylobacter* challenge doses tended to be associated with smaller log reductions. Although this association is borderline at the 5% significance level, it suggests that very high challenge doses may overwhelm probiotic mechanisms such as nutrient competition and antimicrobial secretion, limiting the ability of probiotics to reduce pathogen loads. Experimental studies have similarly reported that probiotics do not always overcome high pathogen colonisation burdens, which supports this interpretation [155,165]. These findings imply that evaluating probiotic efficacy under extremely high experimental challenge doses may underestimate their effectiveness under more realistic field conditions.

Sample type had a strong positive coefficient for caecal samples (1.267 ± 0.029; *p* < 0.001), indicating that, after adjusting for other moderators, the estimated log reduction associated with probiotics was larger when *Campylobacter* concentrations were measured in caecal content rather than in faeces. This is biologically plausible, since, as mentioned before, *Campylobacter* preferentially colonises the caeca, where bacterial densities are typically higher than in faecal material [162,166]. Moreover, the caecal microbiota plays a central role in colonisation resistance; thus, interventions that modulate the caecal microbial community, such as probiotics, may exert more pronounced effects when outcomes are measured at this site [163].

For probiotic interventions, between-study heterogeneity was substantial (I^2^ = 61.8%), and the moderators retained in the model explained about 24% of the between-study variance (R^2^ = 24%). High I^2^ values indicate that much of the variability between studies reflects genuine heterogeneity rather than sampling error [33]. Nevertheless, significant residual heterogeneity remained (QE test, *p* < 0.001), which may partly reflect other study-level differences that could not be included as moderators (e.g., slaughter age, applied dose or age at challenge), due to incomplete reporting in several articles. The test for small-study effects suggested the presence of publication bias (*p* < 0.001; Table 3), indicating that the available evidence on probiotic interventions may over-represent studies reporting larger effects and that pooled estimates should therefore be interpreted with some caution.

#### 3.4.3. Effectiveness of Plant Extracts Against *Campylobacter* Colonisation Levels

The multilevel meta-regression model for plant extracts included slaughter age, treatment duration, applied dose and sample type as moderators (Table 3). Treatment duration had a significant negative coefficient (−0.028 ± 0.012; *p* = 0.012), indicating that the estimated log reduction in *Campylobacter* concentration tended to decrease as the duration of extract treatment increased. In other words, the beneficial effect of plant extracts appeared to be more pronounced during shorter application periods and weakened with longer treatments.

In contrast, both applied dose and slaughter age showed positive and significant associations with the effect of plant extracts (0.121 ± 0.021; *p* < 0.001 and 0.029 ± 0.012; *p* = 0.017, respectively). These results suggest that higher extract concentrations and older slaughter ages were, on average, associated with larger reductions in *Campylobacter* concentration. Biologically, this may reflect a combination of stronger antimicrobial pressure at higher doses and cumulative effects of treatment when birds are exposed for sufficient time before slaughter.

Sample type also influenced the estimated effect of plant extracts. Effect sizes derived from caecal content showed a higher estimated reduction compared with faecal samples (0.121 ± 0.122; *p* = 0.015), and the largest reductions were observed when outcomes were measured in drinking water (0.296 ± 0.169; *p* < 0.001). This pattern is consistent with the fact that the caeca constitute a major reservoir of *Campylobacter* in poultry [162,163], where both pathogen load and microbial interactions are more intense than in faeces, and with the direct exposure of *Campylobacter* present in the drinking system to plant-extract additives. In contrast, faecal samples, which integrate a mixture of intestinal contents and environmental contamination, showed smaller apparent effects of plant extracts on *Campylobacter* concentration.

For plant extracts, between-study heterogeneity was less than moderate (I^2^ = 39.3%); yet, the moderators kept by the model explained a substantial proportion of the between-study variance (R^2^ = 49%). Some residual heterogeneity remained (QE test, *p* < 0.001), which may be partly due to other variables that could not be included as moderators—such as age at application, age at challenge or challenge dose—because of incomplete reporting in several studies. The test for small-study effects did not indicate evidence of publication bias (*p* = 0.499), suggesting that the available estimates for plant-extract interventions are relatively consistent across studies.

#### 3.4.4. Effectiveness of Organic Acids Against *Campylobacter* Colonisation Levels

The multilevel meta-regression model for organic acids included slaughter age, inoculum concentration and application matrix (feed versus drinking water) as moderators (Table 3). All variables showed statistically significant associations with the estimated log reduction in *Campylobacter* concentration (*p* < 0.05). The coefficients for inoculum concentration (0.095 ± 0.054) and slaughter age (0.014 ± 0.004) were positive, indicating that, within the range of values studied, both higher challenge doses and older slaughter ages were associated with greater reductions in *Campylobacter* when organic acids were applied in feed and drinking water.

The application matrix also influenced the intervention effect. Using drinking water as the reference category, the model indicated that delivering organic acids via drinking water was associated with larger log reductions in *Campylobacter* colonisation levels than incorporating them into feed. This is consistent with the hypothesis that administration in water ensures a more uniform distribution and faster passage through the gastrointestinal tract, leading to higher local acid concentrations acting directly on the bacteria [116].

For organic acids, the test for small-study effects did not indicate evidence of publication bias (*p* > 0.05). Between-study heterogeneity was low (I^2^ = 25%), and the moderators retained in the model explained 18% of the between-study variance (R^2^ = 18%). Some residual heterogeneity remained (Q_E test, *p* < 0.001), which may reflect other factors that could not be included as moderators—such as treatment duration, applied dose, age at challenge or route of application—due to limited reporting in several studies.

#### 3.4.5. Effectiveness of Prebiotics Against *Campylobacter* Colonisation Levels

The multilevel meta-regression model for prebiotics included age at application and prebiotic type—fructooligosaccharides (FOS), galactooligosaccharides, mannanoligosaccharides (MOS) and bacitracin methylene disalicylate—as moderators (Table 3). Age at application had a significant effect on the estimated log reduction in *Campylobacter* colonisation levels (*p* = 0.002), indicating that the timing of prebiotic administration influence their performance.

Among the different prebiotic types, MOS showed a small positive coefficient (0.051 ± 0.359), but this effect was not statistically significant (*p* = 0.888), suggesting no clear evidence of a consistent reduction in *Campylobacter* with MOS under the conditions studied. Nevertheless, MOS is known to block pathogen adhesion to the gut epithelium without causing physical damage, which is compatible with a potential protective effect [167,168], although the present data do not allow firm conclusions. In contrast, FOS was associated with a statistically significant negative coefficient (−2.026 ± 0.471; *p* < 0.001), indicating that, relative to the reference category, the use of FOS was linked to smaller reductions—or even higher levels—of *Campylobacter* in treated birds. This unexpected pattern suggests that the interaction between FOS and *Campylobacter* in poultry is complex and may depend on background diet, gut microbiota composition and other management factors [169]. The limited number of studies and differences in study design also call for cautious interpretation of these type-specific effects.

For prebiotic interventions, between-study heterogeneity was negligible (I^2^ = 15.4%), and the moderators included in the model explained most of the between-study variance (R^2^ = 90%). This indicates that age at application and prebiotic type accounted for the majority of the heterogeneity between studies, leaving relatively little residual variability beyond random sampling error [38]. The test for small-study effects did not suggest the presence of publication bias (*p* = 0.348), supporting the robustness of the pooled estimates for prebiotics within the limits of the available data.

The best-fitting multilevel models for chemical treatment, routinary cleaning and disinfection, organic iron complex, non-conventional production systems, vaccination, and transport to slaughter interventions, including the significant moderators retained, the heterogeneity analysis and the publication bias assessment, are summarised in Table 4.

#### 3.4.6. Effectiveness of Chemical Treatments Against *Campylobacter* Colonisation Levels

In this context, “chemical treatments” refers to interventions such as the application of aluminium sulphate, electrolysed oxidising water, bismuth citrate, acidified water and ferric quinic acid complexes either in bird feeding or in treating litter. The multilevel meta-regression model for chemical treatments included slaughter age, sample type (caecal content vs. litter) and application matrix (feed, treated litter and drinking water) as moderators (Table 4). All retained moderators were statistically significant (*p* < 0.001).

Slaughter age was positively associated with the effect of chemical treatments (0.021 ± 0.005), indicating that reductions in *Campylobacter* concentration tended to be greater in older birds at slaughter. With regard to sample type, the negative coefficient for litter relative to caecal content (−1.435 ± 0.096) indicates that, after adjusting for other moderators, chemical treatments appeared less effective when outcomes were measured in litter than when they were measured in caecal content. This is consistent with the idea that *Campylobacter* in caecal content is more directly exposed to the treatment’s effects within the gut, whereas bacteria present in litter may be subject to more heterogeneous exposure conditions and environmental influences [122,170].

Application matrix also influenced the performance of chemical treatments. Using drinking water as the reference category, the model showed that application via feed (0.776 ± 0.089) and treated litter (0.891 ± 0.135) was associated with larger log reductions in *Campylobacter* colonisation in birds and contamination in litters, respectively. The pooled effect on *Campylobacter* colonisation in birds was lower when the chemicals were added to drinking water in comparison to feed. A plausible explanation is that chemical agents act directly on the surfaces of litter and feed, where *Campylobacter* may be more exposed and therefore more susceptible to inactivation [170]. In contrast, *Campylobacter* residing in the caeca is partly protected by the intestinal mucosa, which may limit the impact of some chemical treatments applied only via the environment or water [122].

Between-study heterogeneity for chemical treatments was low (I^2^ = 18.6%), and the moderators included in the model explained more than half the between-study variability (R^2^ = 64%). Nevertheless, significant residual heterogeneity remained (QE test, *p* < 0.001), likely reflecting other study-level differences—such as treatment duration, age at application, challenge dose and age at challenge—that could not be included as moderators due to incomplete reporting. The test for small-study effects indicated evidence of asymmetry compatible with publication bias (*p* = 0.032), suggesting that negative or less favourable results for chemical treatments may be underrepresented in the published literature.

#### 3.4.7. Effectiveness of Routine Cleaning and Disinfection Against *Campylobacter* Colonisation Levels

The multilevel meta-regression model for routine cleaning and disinfection included production system (conventional vs. free-range) and sample type (environmental swabs, litter, floor, soil, drinking water, caecal content and carcasses) as moderators (Table 4). All moderators retained in the model were statistically significant (*p* < 0.05). Overall, routine cleaning and disinfection were associated with larger reductions in *Campylobacter* loads in free-range systems (1.004 ± 0.176) than in conventional systems, suggesting that the same hygiene practices may be more effective—or more strictly implemented—in free-range farms.

The estimated effect also varied by sample type. The largest reductions were observed when *Campylobacter* was quantified in environmental swabs (3.528 ± 0.725 log), followed by drinking water (1.751 ± 0.849 log) and litter (0.422 ± 0.157 low), whereas, at least numerically, smaller effects were found when concentrations were measured in caecal content and on carcasses. This pattern indicates that routine cleaning and disinfection can substantially reduce *Campylobacter* contamination in the farm environment and contact surfaces, but their impact on intestinal colonisation and final carcass contamination is more limited. These findings support the view that cleaning and disinfection alone are insufficient to fully protect birds from *Campylobacter* and must be combined with additional biosecurity and on-farm control measures to effectively reduce public health risks [8,171].

For routine cleaning and disinfection, between-study heterogeneity was low (I^2^ = 30.9%), and the moderators included in the model explained a large proportion of the between-study variance (R^2^ = 66%), although significant residual heterogeneity remained (QE test, *p* < 0.001). The test for small-study effects indicated evidence of asymmetry compatible with publication bias (*p* = 0.009), suggesting that studies reporting weaker or null effects of routine cleaning and disinfection may have not been published.

#### 3.4.8. Effectiveness of Organic Iron Complexes Against *Campylobacter* Colonisation Levels

The multilevel meta-regression model for organic iron complexes included applied dose as a quantitative moderator (Table 4). The slope for dose was highly significant (7.701 ± 1.328; *p* < 0.001) and positive, indicating that higher doses of the organic iron complex were associated with markedly greater log reductions in *Campylobacter* colonisation levels. In other words, within the limited number of available studies, increasing the dose of organic iron complex was linked to stronger reductions in *Campylobacter*, although the magnitude of the estimated coefficient should be interpreted cautiously because of the small dataset and possible differences in how dose was reported.

For organic iron complexes, between-study heterogeneity was low (I^2^ = 11.2%), and the model explained a large proportion of the between-study variance (R^2^ = 72.8%), suggesting that variation in applied dose accounted for most of the heterogeneity between studies. The test for residual heterogeneity was borderline (QE test, *p* = 0.072), indicating that any remaining unexplained between-study variability can be considered negligible. However, the test for small-study effects suggested the presence of asymmetry compatible with publication bias (*p* < 0.001), which, together with the small number of studies, implies that pooled estimates for organic iron complexes should be interpreted with particular caution.

#### 3.4.9. Non-Conventional Production Systems

For the comparison between conventional and non-conventional production systems, the multilevel meta-regression model included sample type as a moderator, with categories comprising environmental swabs, faeces, feed, caecal content, drinking water and carcasses (Table 4). The only association that reached borderline statistical significance (*p* = 0.057) was for environmental swabs, where non-conventional systems tended to show greater reductions in *Campylobacter* contamination than conventional systems. For the other sample types (caecal content, feed, drinking water, faeces and carcasses), the model estimated positive—but non-significant (*p* > 0.10)—coefficients, suggesting a tendency towards lower *Campylobacter* levels in non-conventional systems, although the evidence was not strong enough to draw firm conclusions.

Between-study heterogeneity in this model was very low (I^2^ = 6.5%), and the moderators included explained about 53% of the between-study variance (R^2^ = 53%), indicating that little variability remained beyond what would be expected by chance. The test for small-study effects did not indicate evidence of publication bias (*p* = 0.887), suggesting a relatively consistent pattern of results across the available studies [38].

#### 3.4.10. Effectiveness of Vaccination Against *Campylobacter*

The multilevel meta-regression model for vaccination included slaughter age, treatment duration and route of administration—oral gavage, subcutaneous (SC) and intramuscular (IM)—as moderators (Table 4). Age at application could not be included due to insufficient reporting in the underlying studies. All moderators retained in the model showed statistically significant associations with the estimated log reduction in *Campylobacter* concentration (*p* < 0.10).

Both treatment duration (0.074 ± 0.008) and slaughter age (0.018 ± 0.007) had positive coefficients, indicating that longer vaccination schedules and older slaughter ages were associated, on average, with greater reductions in *Campylobacter* concentration in vaccinated birds. These findings are consistent with the notion that sustained antigen exposure and sufficient time between vaccination and slaughter are needed for the development of a protective immune response [159].

Route of administration also had a marked influence on vaccine performance. Using IM administration as the reference category, the coefficients for oral gavage (−0.305 ± 0.140; *p* = 0.030) and SC administration (−2.009 ± 0.190; *p* < 0.001) were negative, indicating smaller reductions in *Campylobacter* concentration compared with IM vaccination. Thus, among the routes evaluated, IM administration appeared to be the most effective, oral gavage showed intermediate effectiveness, and SC administration was the least effective. These differences are likely related to the distinct immune response profiles elicited by each route and the degree to which they target relevant mucosal sites for *Campylobacter* colonisation [160,172].

For vaccination interventions, the degree of between-study heterogeneity was low (I^2^ = 12%), and the moderators included explained a substantial proportion of the between-study variability (R^2^ = 57.1%). This suggests that differences in slaughter age, treatment duration and administration route accounted for much of the variability in vaccine effects across studies. Nonetheless, the test for small-study effects indicated evidence of asymmetry compatible with publication bias (*p* < 0.001), suggesting that the available evidence may over-represent studies reporting stronger vaccine effects and that pooled estimates should be interpreted with caution.

#### 3.4.11. Transport to Slaughter

The multilevel meta-regression model for transport to slaughter included sample type (caecal content vs. carcasses) as the moderator (Table 4). The effect of sample type was highly significant (*p* < 0.001) and indicated that *Campylobacter* concentrations measured on carcasses were higher than those measured in caecal content. This pattern is consistent with the overall meta-regression, and suggests that transport can contribute to additional load or shedding of *Campylobacter* by birds, particularly increasing the contamination at the skin and feather level. One important factor increasing the risk of contamination during transport is the use of inadequately cleaned or contaminated transport crates [173,174]. Previous studies have reported that *Campylobacter* can survive on crate surfaces for several hours, even after disinfection procedures [175]. Transport crates, reservoirs and vehicles can therefore act as vehicles for transmission between flocks, as supported by findings of multiple *Campylobacter* genotypes on transport equipment, most of which were associated with live chickens and retail chicken meat [175].

For the transport-to-slaughter intervention, between-study heterogeneity was low (I^2^ = 23.3%), and the moderator included in the model explained a large proportion of the between-study variance (R^2^ = 69%), although some residual heterogeneity remained (QE test, *p* = 0.001). The test for small-study effects yielded a borderline *p*-value (*p* = 0.066), suggesting at most weak evidence of asymmetry; overall, there was no strong indication of publication bias for this intervention [36].

### 3.5. Summarisation of the Effects of On-Farm Interventions and Treatments on the Prevalence of Campylobacter in Poultry

To obtain an overview of the effects of on-farm interventions and treatments on the colonisation prevalence of *Campylobacter* in poultry, random-effects meta-regression models were fitted to the complete prevalence data (Table 5).

Effect sizes were expressed as log risk ratios comparing control and treated groups, with positive values indicating lower *Campylobacter* prevalence in the treated flocks. For each intervention category, the overall models estimated the pooled log risk ratio and its standard error, the corresponding *p*-value, and measures of between-study heterogeneity. These pooled estimates presented in Table 5 summarise the average impact of each intervention on *Campylobacter* prevalence across all included studies. Unlike the dataset on concentrations, which was quite extensive, observations on prevalence were available only for six intervention strategies or risk factors, providing complete statistical information for analysis.

**Figure 5:**
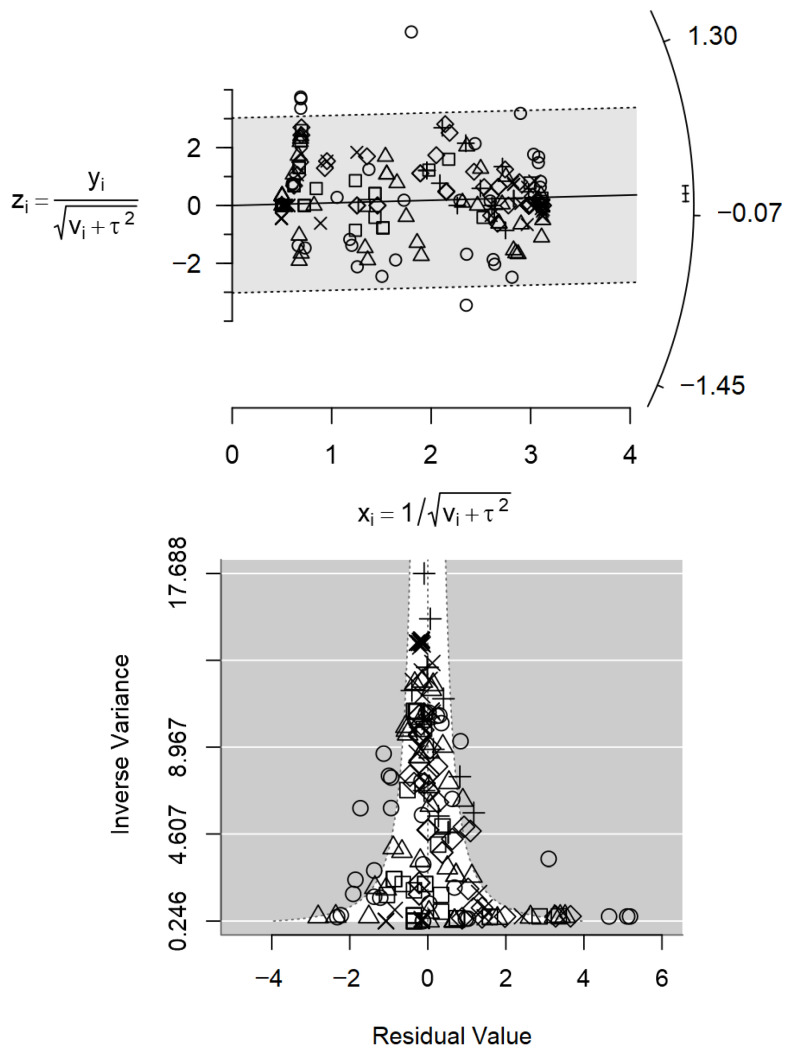
Six-sigma radial plot (**top**) and funnel plot (**bottom**) of the overall meta-regression on the effect of on-farm interventions on the prevalence of *Campylobacter* in poultry. Markers symbolize interventions: □ = *Cleaning and disinfection*, ○ = *Colder climates*, ∆ = *Non-conventional production systems*, + = *Extracts*, × = *Organic acid*, ◊ = *Probiotics*.

Organic acids, colder climates and production system showed positive estimated effects on *Campylobacter* prevalence, but these did not reach statistical significance (*p* > 0.10). In contrast, probiotics, plant extracts and routine cleaning and disinfection were significantly associated with reduced *Campylobacter* prevalence in birds (*p* < 0.05). The largest reduction was observed for probiotics (0.193 ± 0.079), followed by routine cleaning and disinfection (0.119 ± 0.061), while plant extracts showed a smaller, though still significant, effect (0.078 ± 0.036). The Galbraith (radial) plot (Figure 5, top) indicates that most standardised effect sizes fall within the reference bands, with no marked outliers, suggesting a generally consistent pattern across studies. The regression test for small-study effects, comprising the full prevalence data, was not significant (*p* = 0.389; Table 5), and the funnel plot (Figure 5, bottom) appeared approximately symmetrical, providing no clear evidence of publication bias.

Regarding heterogeneity, the overall meta-regression model showed low between-study heterogeneity (I^2^ = 14%), of which about 15% was explained by the types of intervention strategies (R^2^ = 15%). Some residual heterogeneity remained unexplained (QE test, *p* < 0.001), indicating that additional, unmeasured study-level factors may also influence *Campylobacter* prevalence.

### 3.6. Multilevel Meta-Analysis on the Effects of On-Farm Interventions on the Prevalence of Campylobacter in Poultry

The results of the multilevel random-effects meta-regression models fitted separately by on-farm intervention are summarised in Table 6. Across these models, the moderators that showed significant associations with flock prevalence were routine cleaning and disinfection, probiotics, organic acids and plant extracts, although the strength and direction of the associations varied by intervention and by moderator.

#### 3.6.1. Effectiveness of Routine Cleaning and Disinfection Against *Campylobacter* Colonisation Prevalence

The multilevel meta-regression model for routine cleaning and disinfection kept only sample type as a moderator, with categories comprising boot swabs, environmental swabs, faeces, litter, soil, floor, caecal content and water (Table 6). After routine on-farm cleaning and disinfection, significant associations (*p* < 0.10) with reduced *Campylobacter* prevalence were observed in caecal content, in the combined group of floor-related samples (litter, soil and floor), in environmental swabs and in boot swabs. The largest reduction was estimated for the combined litter/soil/floor category (2.475 ± 1.141), followed by boot swabs (0.551 ± 0.369), caecal content (0.235 ± 0.087) and environmental swabs (0.042 ± 0.020). These results suggest that routine cleaning and disinfection can substantially lower *Campylobacter* prevalence on floor surfaces and in the immediate environment, with more modest but still detectable effects at the level of caecal colonisation and environmental swabs. These results aligned well with those previously encountered in the meta-regression of log reduction by routine cleaning and disinfection (i.e., concentration counterpart).

In the case of drinking water and faeces, the pooled effects were not statistically significant. This pattern mirrors the findings for concentration data; and suggests that, in some situations, residual contamination or recontamination of water systems and faecal material may occur despite routine hygiene measures.

For this intervention, between-study heterogeneity was very low (I^2^ = 6.1%), and the moderators included in the model explained about one-third of the between-study variability (R^2^ = 31%), indicating limited variability beyond that expected by chance. The test for small-study effects did not provide evidence of publication bias (*p* = 0.129), supporting a relatively consistent pattern of results across studies [38].

#### 3.6.2. Effectiveness of Probiotics Against *Campylobacter* Colonisation Prevalence

For probiotic interventions, the multilevel meta-regression model included production system (conventional versus other systems), sample type (faeces, caecal content and carcasses) and application matrix (feed, drinking water, oral gavage and spraying) as moderators (Table 6). The overall effect of probiotics on *Campylobacter* prevalence was significantly greater in conventional systems than in other production systems (*p* = 0.004), indicating that, on average, probiotics were more effective at reducing *Campylobacter* prevalence, when flocks were raised under conventional conditions.

Sample type had a marked influence on the estimated effect. Using carcasses as the reference category, negative and significant coefficients were observed for faeces (−1.179 ± 0.477; *p* = 0.014) and caecal content (−1.403 ± 0.463; *p* = 0.003), indicating that the log risk ratio was smaller for these matrices than for carcasses. In practical terms, this means that the apparent impact of probiotics on *Campylobacter* prevalence was weaker—in faeces and caecal samples compared with carcass outcomes. This complements the concentration results, where probiotics showed relatively stronger effects in decreasing colonisation (caecal contents); as, taken together, the meta-regressions suggest that probiotics may have limited impact on reducing the proportion of *Campylobacter*-positive birds within a flock, even if considerable bacterial load reduction is achieved in the colonised birds.

Application route also played an important role. Oral gavage showed the largest and most significant positive association with reduced prevalence (0.865 ± 0.209; *p* < 0.001), indicating that direct oral administration was the most effective mode of delivery among those evaluated. Supplementation via feed also showed a positive and significant effect (0.271 ± 0.133; *p* = 0.041), whereas the effect associated with drinking water was smaller and not clearly distinguishable from the reference. Feed-based delivery is also widely used and actively investigated in antibiotic-free broiler production; for example, Magnoli et al. (2024) evaluated dietary supplementation with *Saccharomyces cerevisiae* var. boulardii RC009 (alone and combined with phytase) in broilers, supporting the practical feasibility of administering probiotics via feed, although the effectiveness on *Campylobacter* levels remains outcome- and context-dependent [176]. Spraying, in contrast, had a near-zero and non-significant coefficient (−0.001 ± 0.068; *p* = 0.991), suggesting that this method was not proven to reduce *Campylobacter* prevalence in chickens.

For probiotics, between-study heterogeneity in prevalence outcomes was very low (I^2^ = 8.75%), and the moderators retained in the model explained a large proportion of the between-study variance (R^2^ = 77%), with no evidence of substantial residual heterogeneity (QE test, *p* = 0.391). However, the test for small-study effects indicated evidence of asymmetry compatible with publication bias (*p* = 0.004), suggesting that studies reporting weaker or adverse effects of probiotic interventions on *Campylobacter* prevalence may be under represented in the published literature [36].

#### 3.6.3. Effectiveness of Organic Acids Against *Campylobacter* Colonisation Prevalence

The multilevel meta-regression model for organic acids included treatment duration and application concentration as quantitative moderators (Table 6). Both variables were statistically significant (*p* < 0.10). Treatment duration showed a small but positive association with the effect of organic acids (0.002 ± 0.001; *p* < 0.001), indicating that longer application periods were associated, on average, with greater reductions in *Campylobacter* prevalence. In contrast, application concentration had a negative coefficient (−0.047 ± 0.018; *p* = 0.009), suggesting that higher proportions of organic acids in feed or drinking water were associated with smaller reductions in prevalence, and in some cases may even have reduced the apparent effectiveness of the intervention. This finding is consistent with the concentration meta-regression results, which indicated that organic acids can be effective in reducing *Campylobacter* when added to drinking water or feed, but that the magnitude of the effect is sensitive to the dose, and excessively high concentrations may not confer additional benefits and could even be detrimental to flock performance or gut ecology.

For prevalence outcomes, between-study heterogeneity in the organic-acid model was high (I^2^ = 64%), and the moderators included explained most of the between-study variance (R^2^ = 95%), indicating that treatment duration and dose accounted for the bulk of the heterogeneity between studies. Some residual heterogeneity remained (QE test, *p* < 0.001), likely reflecting other unmeasured factors such as slaughter age, age at application, age at challenge and challenge inoculum, which could not be incorporated due to insufficient reporting. The test for small-study effects was not significant (*p* = 0.633), indicating no evidence of publication bias and a high degree of consistency across the available studies [38].

#### 3.6.4. Effectiveness of Plant Extracts Against *Campylobacter* Colonisation Prevalence

The multilevel meta-regression model for plant extracts included application concentration as the sole moderator (Table 6). Application concentration was statistically significant and positively associated with the effect of plant extracts on *Campylobacter* prevalence in birds (1.017 ± 0.471; *p* = 0.031), indicating that higher extract concentrations were, on average, linked to greater reductions in flock prevalence. This pattern is consistent with the concentration meta-regression, which also suggested dose-dependent effects of plant extracts on *Campylobacter* levels in poultry.

Between-study heterogeneity in this model was very low (I^2^ = 1.0%), and the moderator explained about 41% of the between-study variance (R^2^ = 41%). Although the QE test indicated some residual heterogeneity (*p* = 0.002), its magnitude was small, and may reflect additional factors that could not be included as moderators. The test for small-study effects was not significant (*p* = 0.504), providing no evidence of publication bias and suggesting that the available results for plant-extract interventions on *Campylobacter* prevalence are generally consistent across studies [41].

### 3.7. Multilevel Meta-Analysis on the Prevalence of Campylobacter in the Farm Environment

To contextualise intervention performance and explain residual heterogeneity, we additionally synthesised *Campylobacter* prevalence across key farm environmental sources that may act as reservoirs and reinfection routes. Detailed source-specific results and discussion are presented in the Supplementary Material [177,178,179].

## 4. Conclusions

This comprehensive systematic review and meta-analysis shows that several non-antibiotic on-farm interventions can reduce the colonisation of *Campylobacter* in poultry flocks, but their effectiveness is strongly context-dependent. For concentration outcomes, the most effective interventions to control *Campylobacter* colonisation in birds were organic acids, plant extracts, probiotics, prebiotics and bacteriophages, whereas prebiotics showed the least consistent and, in some cases, unfavourable effects. Bacteriophage treatments produced modest and largely short-lived reductions, with efficacy declining in older birds and with longer application periods and being sensitive to dose and timing. In contrast, organic acids and plant extracts applied via drinking water or feed showed more robust effects, particularly when appropriately dosed and when outcomes were measured in caecal content rather than faeces. Vaccination effects were influenced by route and schedule, with intramuscular administration outperforming oral and subcutaneous routes, and longer vaccination regimes and sufficient time before slaughter being associated with greater reductions in *Campylobacter* colonisation loads. Routine cleaning and disinfection improved environmental contamination indicators—especially on floors, litter and environmental swabs—but had more limited impact on caecal colonisation and carcass contamination, whereas the stronger apparent effect in free-range than in conventional systems deserves further investigation. Transport to slaughter was associated with increased *Campylobacter* contamination on bird carcasses, highlighting the importance of crate hygiene and transport logistics.

For prevalence outcomes, the most effective interventions were observed when probiotics, plant extracts, and routine cleaning and disinfection were used, while organic acids, colder climates, and production system were inconclusive.

As shown, for both concentrations and prevalence levels of *Campylobacter*, the treatment performance varied with type of intervention and duration, dose and application matrix, mode of administration, age at application and slaughter age, as well as with the sample type used to measure outcomes. In experimentally challenged birds, the pathogen’s dose and age at challenge further influenced the measured effects, underscoring the importance of trial design when evaluating interventions.

Environmental meta-analysis identified deep litter and floor faeces as the main on-farm reservoirs of *Campylobacter*, with intermediate prevalence in sock swabs and air, and lower prevalence in insects. Although insects appear to be a minor reservoir on a percentage basis, their abundance and mobility mean they can still contribute to cross-flock transmission; and therefore, they should not be neglected. Water sources and operator-related samples showed variable but high prevalences, so they represent potential vehicles of spread when biosecurity is inadequate.

Taken together, these findings support the prioritisation of evidence-based, non-antibiotic interventions—such as well-designed feed and water additives, targeted vaccination strategies, and effective cleaning and disinfection—over routine antibiotic use in primary production. Future in vivo trials should more systematically control and report key potential confounders, including bird age, challenge dose, co-infections, production system and detailed intervention protocols, to allow more precise quantification of effects. In addition, for future research we suggest more standardisation reporting in primary studies to reduce heterogeneity.

Overall, this comprehensive meta-analysis study highlighted that no single measure is sufficient to control *Campylobacter* on poultry farms. Meaningful reductions will require a combination of strengthened biosecurity, optimised use of feed and water additives and vaccines, improved litter and environmental management, and careful control of contamination during transport and processing. Integrated, multi-barrier strategies along the farm-to-slaughter continuum remain essential to reduce *Campylobacter* burden at flock level and, ultimately, to lower the risk to public health.

## Figures and Tables

**Table 1:** Overall effects of on-farm interventions and treatments on *Campylobacter* concentration in poultry, based on a random-effects meta-regression model, including pooled log reductions and heterogeneity statistics.

Interventions or Treatments	Log Reduction (Control—Treated)			
	Estimate [SE]	*p*-Value	*n*/*N*	Heterogeneity Analysis
Organic acid in feed/water	0.922 [0.200]	**<0.001**	184/17	I^2^ = 54.3% *p*(QE) < 0.001 R^2^ = 32.0% τ_res_^2^ = 2.289 **Publication bias** *p* < 0.001
Use of bacteriophages	0.837 [0.234]	**<0.001**	98/7	
Extracts in feed/water	0.758 [0.207]	**<0.001**	121/14	
Probiotic in feed/water	0.695 [0.192]	**<0.001**	324/39	
Prebiotic in feed/water	0.694 [0.228]	**0.002**	36/6	
Organic iron complex	**1.599 [0.886]**	**0.071**	12/3	
Chemical treatment in feed/water/litter	**1.086 [0.683]**	0.112	54/5	
Routinary cleaning and disinfection	1.042 [0.879]	0.236	22/3	
Vaccination	0.263 [0.626]	0.674	62/6	
Non-conventional production systems	0.143 [1.079]	0.895	27/2	
Transport to slaughter	−0.497 [0.881]	0.573	24/3	

**Table 2:** Univariate meta-regression analyses * evaluating potential quantitative moderators of the effect of on-farm interventions on *Campylobacter* concentration in poultry.

Quantitative Moderators Tested Individually	Log Ratio of Means (Control Group/Treated Group)					
	Slaughter Age	Duration of Treatment	Age of Application	Application Concentration	Inoculum in Challenge	Age at Challenge
Use of bacteriophages	**0.021** **(−)**	**<0.001** **(−)**	0.795	**0.001** **(−)**	0.119	0.267
Probiotic in feed/water	**0.001** **(−)**	**<0.001** **(+)**	**0.001** **(−)**	**<0.001** **(+)**	**0.023** **(−)**	0.315
Extracts in feed/water	0.158	0.206	0.171	**<0.001** **(+)**	0.210	**0.013** **(+)**
Organic acid in feed/water	**0.022** **(+)**	0.363	0.622	**<0.001** **(+)**	**0.061** **(+)**	0.694
Prebiotic in feed/water	**<0.001** **(+)**	0.290	**<0.001** **(+)**	0.370	0.185	0.115
Chemical treatment in feed/water/litter	**<0.001** **(+)**	**<0.001** **(+)**	**0.049** **(−)**	0.877	NA	0.385
Organic iron complex	NA	NA	NA	**<0.001** **(+)**	NA	NA
Vaccination	**<0.001** **(+)**	**<0.001** **(+)**	0.456	0.960	0.502	**<0.001** **(−)**

(*) For each intervention–moderator combination, the *p*-value for the slope is shown; when significant (α = 0.10), the direction of the association with the moderator is indicated as positive (+) or negative (−). NA: not applicable.

**Table 3:** Best-fit multilevel meta-regression models describing the effects of on-farm interventions ^1^ on *Campylobacter* concentration in poultry.

Intervention	Parameter	Estimate (Log Reduction)	Standard Error	*p*-Value	*n*	Heterogeneity Analysis ^2^
Use of bacterio-phages	Intercept	1.048	0.207	<0.001	36	s^2^ = 0.7240 τ^2^ = 0.1194 I^2^ = 14.2% *p*(QE) < 0.001 R^2^ = 0.432 Pub. bias *p* = 0.407
	Treatment duration	−0.021	0.005	0.001		
	Age of application	0.015	0.005	0.017		
	Application concentration	−0.041	0.010	<0.001		
	Sample type					
	Caecal	0.386	0.161	0.017		
	Faeces	-	-	-		
Probiotics in feed/water	Intercept	1.613	1.055	0.127	234	s^2^ = 2.754 τ^2^ = 4.457 I^2^ = 61.8% *p*(QE) < 0.001 R^2^ = 0.239 Pub. bias *p* < 0.001
	Treatment duration	0.012	0.002	<0.001		
	Age of application	−0.025	0.009	0.007		
	Inoculum concentration	−0.319	0.168	0.057		
	Sample type					
	Caecal	1.267	0.029	<0.001		
	Faeces	-	-	-		
Extracts in feed/water	Intercept	−0.100	0.327	0.759	101	s^2^ = 0.9568 τ^2^ = 0.6209 I^2^ = 39.3% *p*(QE) < 0.001 R^2^ = 0.486 Pub. bias *p* = 0.499
	Slaughter age	0.029	0.012	0.017		
	Treatment duration	−0.028	0.012	0.012		
	Application concentration	0.121	0.021	<0.001		
	Sample type					
	Caecal	0.121	0.122	0.015		
	Water	0.296	0.169	<0.001		
	Faeces	-	-	-		
Organic acids in feed/water	Intercept	−0.018	0.341	0.957	103	s^2^ = 0.7118 τ^2^ = 0.2359 I^2^ = 24.9% *p*(QE) < 0.001 R^2^ = 0.182 Pub. bias *p* = 0.405
	Slaughter age	0.014	0.004	0.001		
	Inoculum concentration	0.095	0.054	0.079		
	Application matrix					
	Feed	−0.892	0.053	<0.001		
	Drinking water	-	-	-		
Prebiotics in feed/water	Intercept	−0.069	0.183	0.704	19	s^2^ = 1.030 τ^2^ = 0.1875 I^2^ = 15.4% *p*(QE) = 0.260 R^2^ = 0.901 Pub. bias *p* = 0.348
	Age of application	0.018	0.006	0.002		
	Source					
	Fructooligosaccharide	−2.026	0.471	<0.001		
	Galactooligosaccharide	−0.077	0.282	0.785		
	Mannanoligosaccharide	0.051	0.359	0.888		
	Bacitracin methylene disalicylate	-	-	-		

^1^ For each intervention, the table reports the number of observations (*n*), the significant moderators retained in the final model, measures of between-study heterogeneity, and the *p*-value of the test for small-study effects (publication bias). ^2^ Heterogeneity analysis encompasses within-study variability (s^2^), between-study variability (τ^2^), and intra-class correlation (I^2^) of the null model, and QE test of residual heterogeneity, and square-root of the between-study variability explained by significant moderators (R^2^) from the full model.

**Table 4:** Best-fit multilevel meta-regression models describing the effects of on-farm interventions ^1^ on *Campylobacter* concentration in poultry.

Intervention	Parameter	Estimate (Log Reduction)	Standard Error	*p*-Value	*n*	Heterogeneity Analysis ^2^
Chemical treatment in feed/water/litter	Intercept	0.632	0.231	0.006	48	s^2^ = 0.5939 τ^2^ = 0.1355 I^2^ = 18.6% *p*(QE) < 0.001 R^2^ = 0.644 Pub. bias *p* = 0.032
	Slaughter age	0.021	0.005	<0.001		
	Sample type					
	Caecal	−1.435	0.096	<0.001		
	Litter	-	-	-		
	Application matrix					
	Feeding	0.776	0.089	<0.001		
	Litter treatment	0.891	0.135	<0.001		
	Drinking water	-	-	-		
Routinary cleaning and disinfection	Intercept	−1.081	0.418	0.010	19	s^2^ = 2.1780 τ^2^ = 0.9769 I^2^ = 30.9% *p*(QE) < 0.001 R^2^ = 0.662 Pub. bias *p* = 0.009
	Production system					
	Free-range	1.004	0.176	<0.001		
	Conventional	-	-	-		
	Sample type					
	Environment swabs	3.528	0.725	<0.001		
	Litter, floor, soil	0.422	0.157	0.007		
	Water	1.751	0.849	0.039		
	Caecal/carcass	-	-	-		
Organic iron complex	Intercept	0.863	0.264	0.001	12	s^2^ = 0.6983 τ^2^ = 0.0877 I^2^ = 11.2% *p*(QE) = 0.072 R^2^ = 0.728 Pub. bias *p* < 0.001
	Application concentration	7.701	1.328	<0.001		
Non-conventional production systems	Intercept	−0.048	0.303	0.873	27	s^2^ = 0.6576 τ^2^ = 0.0455 I^2^ = 6.5% *p*(QE) = 0.210 R^2^ = 0.530 Pub. bias *p* = 0.887
	Sample type					
	Environment swabs	0.536	0.297	0.057		
	Faeces	0.034	0.325	0.917		
	Feed	0.132	0.305	0.666		
	Caecal	0.144	0.420	0.732		
	Water	0.354	0.304	0.245		
	Carcass	-	-	-		
Vaccination	Intercept	−0.588	0.243	0.016	52	s^2^ = 1.3575 τ^2^ = 0.1858 I^2^ = 12.0% *p*(QE) = 0.980 R^2^ = 0.571 Pub. bias *p* < 0.001
	Slaughter age	0.018	0.007	0.013		
	Treatment duration	0.074	0.008	<0.001		
	Application					
	Oral gavage	−0.305	0.140	0.030		
	Subcutaneous	−2.009	0.190	<0.001		
	Intramuscular	-	-	-		
Transport to slaughter	Intercept	−0.238	0.211	0.272	23	s^2^ = 0.8918 τ^2^ = 0.2703 I^2^ = 23.3% *p*(QE) = 0.001 R^2^ = 0.692 Pub. bias *p* = 0.066
	Sample type					
	Carcass	−1.000	0.187	<0.001		
	Caecal	-	-	-		

^1^ For each intervention, the table reports the number of observations (*n*), the significant moderators retained in the final model, measures of between-study heterogeneity, and the *p*-value of the test for small-study effects (publication bias). ^2^ Heterogeneity analysis encompasses within-study variability (s^2^), between-study variability (τ^2^), and intra-class correlation (I^2^) of the null model, and QE test of residual heterogeneity, and square-root of the between-study variability explained by significant moderators (R^2^) from the full model.

**Table 5:** Overall effects of on-farm interventions on the prevalence of *Campylobacter* in poultry, showing heterogeneity analysis of the meta-regression.

Interventions or Treatments	Log of Risk Ratio (Control Group/Treated Group)			
	Estimate [SE]	*p*-Value	*n*/*N*	Heterogeneity Analysis ^1^
Probiotics	0.193 [0.079]	**0.014**	46/7	I^2^ = 14.0% *p*(Q) < 0.001 R^2^ = 15.7% τ_res_^2^ = 0.2412 **Pub. bias** *p* = 0.389
Extracts in feed/water	0.078 [0.036]	**0.027**	15/2	
Routinary cleaning and disinfection	0.119 [0.061]	**0.054**	39/4	
Organic acid in feed/water	0.158 [0.206]	0.443	76/4	
Colder climates (versus warmer)	0.195 [0.289]	0.499	32/4	
Non-conventional production Systems (versus conventional)	0.207 [0.454]	0.971	43/4	

^1^ Heterogeneity analysis encompasses within-study variability (s^2^), between-study variability (τ^2^), and intra-class correlation (I^2^) of the null model, and QE test of residual heterogeneity, and square-root of the between-study variability explained by significant moderators (R^2^) from the full model.

**Table 6:** Best-fitting multilevel meta-regression models describing the effects of on-farm interventions ^1^ on the prevalence of *Campylobacter* in poultry.

Intervention	Parameter	Estimate	Standard Error	*p*-Value	*n*	Heterogeneity Analysis ^2^
Routinary cleaning and disinfection	Sample type				39	s^2^ = 0.9247 τ^2^ = 0.0600 I^2^ = 6.1% *p*(QE) = 0.801 R^2^ = 0.311 Pub. bias *p* = 0.129
	Boots	0.551	0.369	0.137		
	Environment swabs	0.042	0.020	0.039		
	Faeces	−0.141	0.355	0.691		
	Litter, soil, floor	2.475	1.141	0.030		
	Caecal	0.235	0.087	0.001		
	Water	−0.141	0.434	0.745		
Probiotics	Intercept	0.656	0.513	0.201	44	s^2^ = 0.3211 τ^2^ = 0.0308 I^2^ = 8.75% *p*(QE) = 0.391 R^2^ = 0.769 Pub. bias *p* = 0.004
	Production system					
	Conventional	0.516	0.179	0.004		
	Others	-	-	-		
	Sample type					
	Faeces	−1.179	0.477	0.014		
	Caecal	−1.403	0.463	0.003		
	Carcass	-	-	-		
	Application					
	Feeding	0.271	0.133	0.041		
	Oral gavage	0.865	0.209	<0.001		
	Spraying	−0.001	0.068	0.991		
	Drinking water	-	-	-		
Organic acids in feed/water	Intercept	2.343	0.900	0.009	39	s^2^ = 0.2940 τ^2^ = 0.5136 I^2^ = 63.6% *p*(QE) < 0.001 R^2^ = 0.951 Pub. bias *p* = 0.633
	Duration of treatment	0.002	0.001	<0.001		
	Application concentration	−0.047	0.018	0.009		
Extracts	Intercept	−0.101	0.079	0.201	13	s^2^ = 0.1861 τ^2^ = 0.0018 I^2^ = 1.00% *p*(QE) = 0.002 R^2^ = 0.408 Pub. bias *p* = 0.504
	Application concentration	1.017	0.471	0.031		

^1^ For each intervention, the table reports the number of observations (*n*), the significant moderators retained in the final model, measures of between-study heterogeneity, and the *p*-value of the test for small-study effects (publication bias). ^2^ Heterogeneity analysis encompasses within-study variability (s^2^), between-study variability (τ^2^), and intra-class correlation (I^2^) of the null model, and QE test of residual heterogeneity, and square-root of the between-study variability explained by significant moderators (R^2^) from the full model.

## Data Availability

No new data were created or analysed in this study. Data sharing is not applicable to this article.

## Supplementary Materials

The following supporting information can be downloaded at https://www.mdpi.com/article/10.3390/foods15020307/s1, File S1: Multilevel meta-analysis on the prevalence of *Campylobacter* in the farm environment.
