# Supplementary material for: Effectiveness of Interventions and Control Measures in the Reduction of Campylobacter in Poultry Farms: A Comprehensive Meta-Analysis
Source: Foods. 2026 Jan 14;15(2):307. doi: 10.3390/foods15020307 (PMC12840304; doi:10.3390/foods15020307)
Supplement: Supplementary file 1 [file foods-15-00307-s001.zip › foods-4039586-supplementary.pdf]

Supplementary Material 1: Multilevel meta-analysis on the prevalence of *Campylobacter* in the farm environment

To identify the main on-farm reservoirs and risk factors for the persistence and spread of *Campylobacter*, a multilevel random-effects meta-analysis was performed using prevalence data from live birds and environmental samples. Environmental sources included litter, floor faeces, boot socks, drinkers, feeders, air, insects and other matrices, as reported in the original studies. Prevalence estimates for each source were modelled on the logit scale and then back-transformed to obtain pooled proportions.

The pooled prevalence estimates and corresponding heterogeneity statistics for each animal or environmental source are summarised in Table S1.

The overall meta-regression showed that, among the environmental sources with statistically significant prevalence ($p < 0.001$), deep litter was the leading on-farm reservoir of *Campylobacter*, with the highest pooled prevalence (19.3%; 95% CI: 10.0–34.0%). Other authors have reported similarly high contamination levels, with prevalence reaching 100% in litter and over 80% in caecal content and have identified litter as a major risk factor for the spread of *Campylobacter* in primary poultry production, particularly when litter is reused or inadequately treated between flocks, or when downtime between flocks is insufficient [177]. Floor faeces were the second most important reservoir, with a pooled prevalence of 16.8% (95% CI: 9.6–22.7%), confirming faeces as a central source with high potential for within- and between-flock transmission of *Campylobacter* [141].

Table S1. Pooled prevalence of *Campylobacter* sampled from live animals and environmental elements in poultry farms, showing heterogeneity analysis

Source	Pooled prevalence	95% CI	p-value	n/N	Heterogeneity Analysis[1]
Air	0.081	[0.045 – 0.140]	**<0.001**	7/2	
Deep litter	0.193	[0.100 – 0.340]	**<0.001**	16/7	I^2=15.5%
Floor faeces	0.168	[0.096 – 0.277]	**<0.001**	41/4	p(Q) < 0.001
Insects	0.031	[0.009 – 0.098]	**<0.001**	7/4	R^2=27.8%
Live birds (cloaca)	0.351	[0.190 – 0.556]	0.152	86/11	τ_{res}^2=1.659
Miscellaneous	0.167	[0.107 – 0.253]	**<0.001**	68/9	**Publication bias**
Operators	0.493	[0.315 – 0.672]	0.937	10/3	P<0.001
Socks	0.086	[0.045 – 0.158]	**<0.001**	14/3	
Water	0.426	[0.133 – 0.782]	0.708	3/2	

[1]Heterogeneity analysis encompasses within-study variability (s^2), between-study variability (τ^2), and intra-class correlation (I^2) of the null model, and QE test of residual heterogeneity, and square-root of the between-study variability explained by significant moderators (R^2) from the full model

For miscellaneous environmental elements - such as swabs from boards, boots, box lines, breast supports, transport crates, drinkers, feeders, ventilation systems and walls - the pooled prevalence was 16.7% (95% CI: 11.0–25.0%). This is in line with other systematic reviews [174,178], which reported *Campylobacter* prevalence values typically ranging from 10% to 25% in these matrices and highlighted their role in persistence and transmission during and between production cycles. Sock swabs (8.6%; 95% CI: 5.0–16.0%) and air samples (8.1%; 95% CI: 5.0–14.0%) showed intermediate prevalence. This likely reflects the ability of sock swabs to capture contamination from floors and litter, while air and

dust particles can harbour bacteria, especially in high-density barns with suboptimal ventilation [138].

The lowest pooled prevalence was observed in insects (3.1%; 95% CI: 1.0–5.6%). Although this meta-analysis suggests that insects are not the primary reservoir for *Campylobacter* on farms, their large numbers and mobility mean that they can still contribute to cross-contamination between flocks [174]. Overall, these findings emphasise the importance of reinforcing biosecurity and hygiene measures targeting litter management, floor and environmental contamination, and vector control to minimise *Campylobacter* prevalence in poultry farms.

In this meta-analysis, operators (49.3%; 95% CI: 31.5 – 67.2%) and water (including puddles, nipple drinkers, and coop rinse water; 42.6%; 95% CI: 13.3 – 78.2%) showed relatively high pooled prevalence estimates, and the non-significant p-values for these sources should be interpreted as simple heterogeneity between studies. The prevalence of *Campylobacter*-positive live birds was estimated at 35.1% (95% CI: 19.0 – 55.6%); similar order of magnitude as operators and water/drinkers. Operators can, in principle, introduce and disseminate *Campylobacter* through contaminated hands, boots or clothing, especially when biosecurity measures are weak. However, previous analyses have reported that, although isolated samples from operators may show high prevalence, the overall contribution of operator-related contamination to flock-level *Campylobacter* status is often not statistically significant once confounding factors such as flock density and environmental conditions are accounted for. Similarly, puddles, drinkers and coop rinse water can be contaminated with *Campylobacter*, but routine monitoring tends to yield highly variable results, and meta-analyses generally do not find a consistent, statistically significant association between these water sources and flock colonisation [179].

These source-specific prevalence patterns are consistent with reinfection pressure within houses and support combining targeted microbiological interventions with measures addressing litter management, water hygiene, and vector control as part of a multi-barrier approach.

The analysis of small-study effects provided strong evidence of asymmetry compatible with publication bias ($p < 0.001$), suggesting that relevant studies may be missing from the pooled dataset. This was corroborated by the asymmetry observed in the funnel plot (Figure S1, bottom). Despite this, the Galbraith plot (Figure S1, top) showed that standardised effect sizes were broadly distributed around the regression line across the range of precisions, with no extreme outliers.

With regard to heterogeneity, the model showed low between-study heterogeneity ($I^2 = 15.5\%$), of which about 27.8% was explained by the sources ($R^2 = 27.8\%$). Some residual heterogeneity remained unexplained, indicating that additional, unmeasured study-level factors may also influence *Campylobacter* prevalence in these environmental and operator-related sources.

Table S2 summarises how rearing time influences the prevalence of *Campylobacter* in live birds and environmental samples. The meta-regression showed that, as rearing time increased, *Campylobacter* prevalence tended to rise in floor faeces, miscellaneous environmental samples, sock swabs and live birds (Table S2, Figure S2). In contrast, a decreasing trend in prevalence with increasing rearing time was observed in deep litter, suggesting that litter dynamics over the production cycle may differ from those of other environmental sources.

In this analysis, between-study heterogeneity was low ($I^2 = 28.5\%$; Table S2), and the moderators included in the model explained just over half of the between-study variance ($R^2 > 50\%$). Some residual heterogeneity remained unexplained (QE test, $p < 0.001$), indicating that additional factors not captured in the model may also affect prevalence patterns over time. The test for small-study effects provided strong evidence of asymmetry

compatible with publication bias ($p < 0.001$), suggesting that studies with weaker or null time-related effects may be underrepresented in the available literature [36].

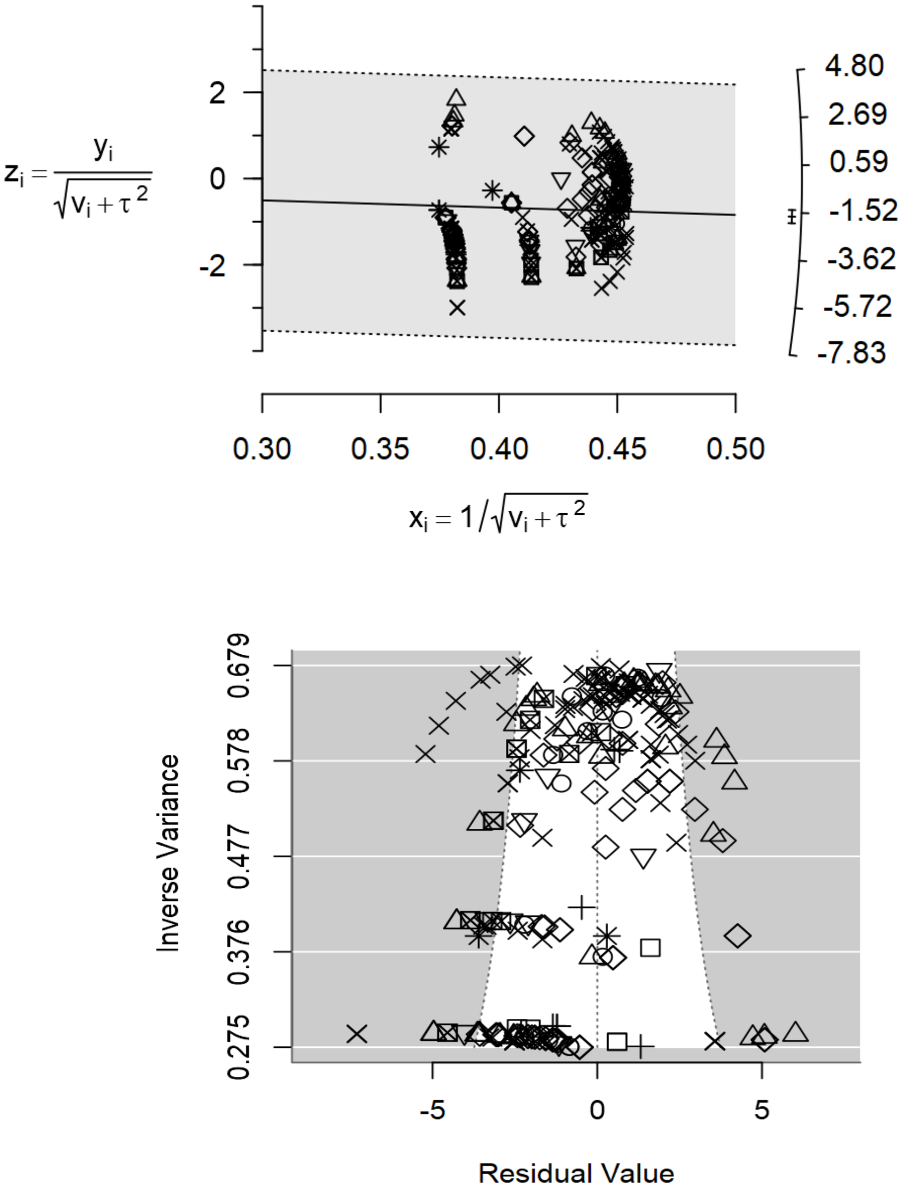

Figure S1. Six-sigma radial plot (top) and funnel plot (bottom) of the meta-analysis of prevalence of *Campylobacter* sampled from live animals and environmental elements in poultry farms. Markers symbolize sample sources: □= Air, ○= Deep litter, Δ= Floor faeces, += Insects, ×= Live birds (cloaca), ◊= Miscellaneous elements, ▼= Operators, ⊠= Socks, * = Water

Table S2. Effect of rearing time (day) on the logit of prevalence of *Campylobacter* in live animals and environmental elements in poultry farms. Number of observations (n), heterogeneity analysis, and p-value of the publication bias test are shown

Parameter	Estimate	Standard error	p-value	n	Heterogeneity analysis[1]
Source					$s^2 = 7.2107$
Deep litter	-2.084	1.272	0.101		$\tau^2 = 1.6425$
Floor faeces	-2.818	0.582	<0.001		$I^2 = 28.5\%$
Live birds (cloaca)	-2.442	0.385	<0.001		$p(QE) < 0.001$
Miscellaneous	-3.019	0.773	<0.001		$R^2 = 0.529$
Socks	-5.762	0.623	<0.001	158	Pub. bias
Time					$p < 0.001$
Deep litter	0.002	0.047	0.974		
Floor faeces	0.083	0.008	<0.001		
Live birds (cloaca)	0.037	0.004	<0.001		
Miscellaneous	0.067	0.010	<0.001		
Socks	0.063	0.022	0.004		

[1]Heterogeneity analysis encompasses within-study variability (s^2), between-study variability (τ^2), and intra-class correlation (I^2) of the null model, and QE test of residual heterogeneity, and square-root of the between-study variability explained by significant moderators (R^2) from the full model

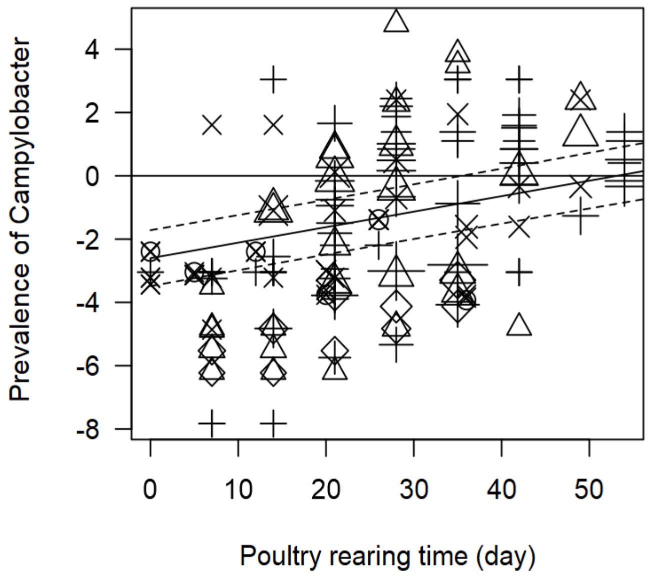

Figure S2. Meta-analytical bubble plot depicting the impact (*p*<0.01) of poultry rearing time on the prevalence of *Campylobacter* in environmental elements and animals on farms. Markers symbolize sample sources: ○= Deep litter, Δ= Floor faeces, += Live animals (cloaca), ×= Miscellaneous elements, ◊= Socks
